# Use of a random coefficient regression (RCR) model to estimate growth parameters

**DOI:** 10.1186/1471-2156-4-S1-S5

**Published:** 2003-12-31

**Authors:** Jonathan Corbett, Aldi Kraja, Ingrid B Borecki, Michael A Province

**Affiliations:** 1 Division of Biostatistics, Washington University School of Medicine, 660 South Euclid Avenue, St. Louis, Missouri, USA

## Abstract

We used a random coefficient regression (RCR) model to estimate growth parameters for the time series of observed serum glucose levels in the Replicate 1 of the Genetic Analysis Workshop 13 simulated data. For comparison, a two time-point interval was also selected and the slope between these two observations was calculated. This process yielded four phenotypes: the RCR growth phenotype, a two time-point slope phenotype, and Time 1 and Time 2 serum glucose level phenotypes. These four phenotypes were used for linkage analyses on simulated chromosomes 5, 7, 9, and 21, those chromosomes that contained loci affecting the growth course for serum glucose levels. The linkage analysis of the RCR-derived phenotype showed overwhelming evidence for linkage at one locus (LOD 65.78 on chromosome 5), while showing elevated but nonsignificant LOD scores for two other loci (LOD 1.25 on chromosome 7, LOD 1.10 on chromosome 9), and no evidence of linkage for the final locus. The two time-point slope phenotype showed evidence for linkage at one locus (LOD 4.16 on chromosome 5) but no evidence for linkage at any of the other loci. A parallel cross-sectional approach, using as input phenotypes the endpoints of the two-point slope phenotype, gave strong linkage results for the major locus on chromosome 5 (maximal LOD scores of 17.90 and 27.24 for Time 1 and Time 2, respectively) while showing elevated but nonsignificant linkage results on chromosome 7 (maximal LOD scores of 1.71 and 1.48) and no evidence for linkage at the two remaining loci. The RCR growth parameter showed more power to detect linkage to the major locus than either the cross-sectional or two-point slope approach, but the cross-sectional approach gave a higher maximal LOD score for one of the minor loci.

## Background

Longitudinal studies are often designed to investigate the progression of a trait over time by taking repeated measurements in each study participant. For many traits, there are assumed to be genetic influences not only upon baseline values, but also on the course of the trait over time. If individual growth curves share the same basic model with parameters varying by individual, e.g., if a trait varies linearly over time with the slope and intercept varying between individuals, then we might estimate growth parameters from longitudinal data using a random coefficient regression (RCR) model [1,2].

This analysis used an RCR model to estimate growth curve parameters for the glucose trait in Replicate 1 of the Genetic Analysis Workshop 13 (GAW13) simulated data with answers known at the time of analysis. The RCR model was implemented in SAS using the PROC MIXED procedure. The resulting parameters were then used as phenotypes for a variance-components based linkage analysis method implemented in the computer software package SEGPATH [3]. While the GAW13 simulated data are nearly ideal for examination using the RCR method, it is uncommon to have measurements of a phenotype at 21 time points. Thus, we also performed parallel linkage analyses of the data using only two time points.

## Methods

The RCR model is a two-stage model which admits both individual-level and population-level effects. It is known to be robust against data that are not missing completely at random [1].

Let y be a trait measured at *n *time points in *m *individuals, yielding *m *time series of *n *measurements each: *y*_*ij *_(*i *= 1, ..., *n *; *j *= 1, ..., *m*). Note that some *y*_*ij *_may be missing. Let us denote by *y*_*j *_the time series of observations for the *j*^th ^participant. Population level effects, i.e., covariates which are assumed to affect the trait *y *in the same manner for all subjects, are modeled by *C*, an *n *× *p *matrix of regressors. The *j*^th ^participant's values for these *p *covariates are given by a *p *× 1 data vector, ξ_*j *_(*j *= 1, ..., *m*).

Individual-level effects, that is the effects of covariates which may impact each subject differently, are modeled by a family of *q *× 1 data vectors, ζ_*j *_(*j *= 1, ..., *m*), consisting of the subjects observed values for the *q *individual level covariates, and *n *× *q *matrices of regressors, *B*_*j*_. Thus, there are different matrices of regressors (*B*_*j*_) for each individual. An example of an individual level effect would be a linear dependence of a trait on age with slope and intercept values that vary from individual to individual. The conditional expected value of the time series is then a sum of the population level and individual level effects:

*E*(*y*_*j *_| ξ_*j*_, ζ_*j*_) = *C *ξ_*j *_+ *B*_*j *_ζ_*j*_.

We make a homoscedasticity assumption in that we assume that the conditional variance of the time series, *V *= Var(*y*_*j *_| ξ_*j*_, ζ_*j*_), depends on the individual only through the number of, and time between, observations. We assume further that observations, while being correlated within individuals, are independent between individuals and that the distribution of the conditional time series is multivariate normal.

For the GAW13 simulated data, many possible RCR models were tested on the time series of observations of the serum glucose phenotype. The RCR modeling framework assumes that the individual level parameters, i.e., those coefficients of the individual level regressor matrices *B*_*j *_which are variable, are drawn from a joint multivariate normal distribution. Individual level covariance structures tested included linear, quadratic, and exponential models of age dependence. We shall hereafter refer to the estimates of the regression coefficient(s) in the individual level effects that correspond to age dependence as the growth or slope parameter(s).

Population-level covariance structures tested included those with and without body mass index (BMI) and sex effects. Note that these models, while differing in the number of covariates or particular structure assigned to the individual and/or population levels of variance effects, all take the same basic form within the framework of the RCR model paradigm. Models were selected based upon their fit as quantified by the Akaike Information Criterion (AIC). The modeling and fitting was performed in SAS using the PROC MIXED procedure.

For comparison, a two time-point subset of the sample was selected by taking the first and third observations in the first cohort (constituting a four-year interval) and the first and second observations in the second cohort (constituting a five-year interval). The slope between these observations was then used as an alternative growth parameter phenotype. In addition, the glucose levels at each of the two time points in this subset were separately analyzed as phenotypes. This cross-sectional approach serves as a contrast with the linkage analysis using the growth parameter(s).

All four phenotypes (Time 1 and 2 glucose levels, two-point slope, and the slope parameter(s) from the RCR model) were used as input phenotypes for a linkage analysis in SEGPATH. SEGPATH performs variance-components linkage analysis on complex, extended pedigrees [3,4].

## Results

The RCR model selected was an exponential growth model (with one parameter) at the individual level. In particular the *B*_*j *_and ζ_*j *_were selected to be of the form:



so that the expected value of the log of the glucose level (denoted by *y*) for a subject at age *t *(after regressing out population level effects) would be assumed to be given by:

*E *(*y*) = *B*_*j *_ζ_*j *_= α_*j*_*t *+ β_*j*_.

Furthermore, α_*j *_and β_*j *_are assumed to follow a bivariate normal distribution.

At the population level, following our rule to include or exclude covariates based upon the AIC of examined models, sex was included as a covariate while BMI was excluded. The individual growth parameter α_*j *_was then used as a phenotype for linkage analysis. This analysis was performed on chromosomes 5, 7, 9, and 21, the chromosomes which contained the QTLs that affected the growth curve for glucose levels.

**Figure 1:**
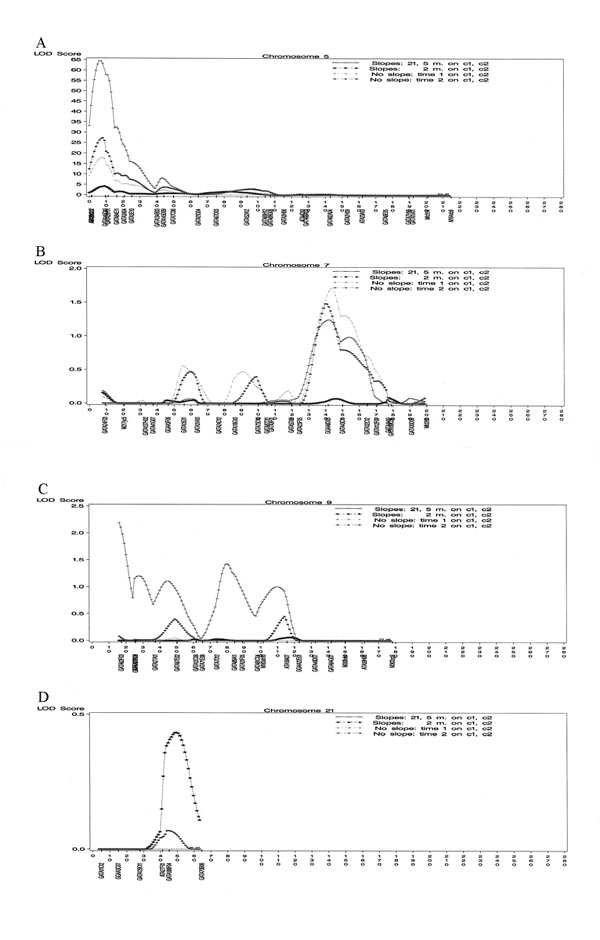
GAW13 LOD score plots for glucose slopes on chromosome 5 (A), chromosome 7 (B), chromosome 9 (C), and chromosome 21 (D).

One region was found to be linked significantly to the RCR model growth parameter phenotype. The peak was on chromosome 5 at 9 cM with a LOD score of 65.78 (Figure 1A). This phenotype also showed elevated LOD scores at 142 cM on chromosome 7 (LOD 1.25) (Figure 1B) and at 45 cM on chromosome 9 (LOD 1.10) (Figure 1C), but these LOD scores fall short of genome-wide statistical significance. No linkage was found to the locus on chromosome 21 (Figure 1D).

In contrast, the two-point slope phenotype gave only one significant region of linkage on chromosome 5 (LOD 4.16 at 9 cM). The two single-point phenotypes gave significant evidence for linkage to chromosome 5 (LOD scores of 17.90 and 27.24 at 9 cM for Time 1 and Time 2, respectively) and elevated LOD scores on chromosome 7 (maximal LOD scores of 1.71 and 1.48 at 141–144 cM.) Table 1 contains the highest LOD values observed for each phenotype within 20 cM of the true location of the locus.

## Discussion

The model selected by PROC MIXED in SAS closely resembled the simulated disease model. The linear and quadratic models were correctly rejected in favor of an exponential growth model, and sex was correctly included as a population-level fixed effect. However, despite influencing the trait on a population level, BMI was not included as a population-level covariate based upon the AIC scores for the models, which included it. The model selection process did not appear to be affected by the presence of missing phenotypic data or by the difference in sampling procedures between the first and second cohorts.

Of the four chromosomal regions linked to the growth course of serum glucose levels that were modeled, only one was discovered unambiguously. However, the one true signal discovered was discovered with an overwhelming LOD score (LOD 65.78). Two of the remaining three regions (on chromosomes 7 and 9) showed elevated but nonsignificant LOD scores, while one (on chromosome 21) showed no evidence for linkage to the RCR growth curve parameter.

Evidence for linkage was present in the two time-point slope phenotype only in chromosome 5. The LOD score, while respectable (LOD 4.16), did not come close to matching the LOD score for the RCR growth parameter. The two-point slope phenotype showed no evidence for linkage at any of the other chromosomal regions simulated to affect the growth course of glucose levels. The LOD scores in those regions were well below the LOD scores found using the RCR growth parameter.

The RCR growth parameter thus proved much more powerful at discovering the location of loci affecting the growth curve for serum glucose levels than the two time-point slope phenotype. Not only did the RCR model virtually recover the model used to simulate the glucose level time series, but it had the advantage over the two time-point slope phenotype of incorporating information from all of the recorded observations. However, the two single time-point phenotypes proved capable not only of detecting linkage to the major gene on chromosome 5, but also gave higher LOD scores than the RCR growth parameter for the minor locus on chromosome 7.

The relatively high LOD scores provided by the two single-point phenotypes indicates, especially vis-à-vis the two-point slope phenotype, the utility of a cross-sectional approach to the detection of genes affecting growth courses, at least in cases such as this simulation, where a difference in means is easily distinguishable. In this simulation, genes affecting the growth of glucose levels caused large divergences beginning at birth. Had these genes been coupled with low initial glucose levels, for example, in order that their effects not been so easily detected, a cross-sectional approach might not have been so fruitful. In this case, we may be seeing an effect in which the differing means between groups of individuals with different growth course affecting genotypes at a given age are more easily seen and are less affected by noise and error than differing slopes for those groups, at least until a large number of measurements are available to estimate the growth course.

## Figures and Tables

**Table 1:** Maximal LOD scores and locations for each locus, compared with true location for each locus

**Phenotypes**	**Chr. 5 (8.46 cM)**	**Chr. 7 (130.84 cM)**	**Chr. 9 (58.02 cM)**	**Chr. 21 (9.04 cM)**
RCR Growth Curve Parameter	65.78 (9 cM)	1.25 (142 cM)	1.10 (45 cM)	0.00
Two-Point Slope	4.16 (9 cM)	0.07 (145 cM)	0.03 (61 cM)	0.00
Time 1, Single-Point	17.90 (9 cM)	1.71 (144 cM)	0.05 (49 cM)	0.00
Time 2, Single-Point	27.24 (9 cM)	1.48 (141 cM)	0.40 (49 cM)	0.00

## References

[B1] Laird NM, Ware JH (1982). Random effects models for longitudinal data. Biometrics.

[B2] Rutter CM, Elashoff RM (1994). Analysis of longitudinal data: random coefficient regression modeling. Stat Med.

[B3] Province MA, Rao DC (1995). A general purpose model and a computer program for combined segregation and path analysis (SEGPATH): automatically creating computer programs from symbolic language model specifications. Genet Epidemiol.

[B4] Province MA, Rice TK, Borecki IB, Gu C, Kraja A, Rao DC (2003). A multivariate and multilocus variance components method based upon structural relationships to assess quantitative trait linkage via SEGPATH. Genet Epidemiol.

